# Supplementary material for: Physiologically based pharmacokinetic/pharmacodynamic model for the prediction of morphine brain disposition and analgesia in adults and children
Source: PLoS Comput Biol. 2021 Mar 4;17(3):e1008786. doi: 10.1371/journal.pcbi.1008786 (PMC7963108; doi:10.1371/journal.pcbi.1008786)
Supplement: Attachment — Submitted filename: Verscheijden. Response to reviewers.docx [file pcbi.1008786.s007.docx]

**Reviewer #1: Major**

*We would like to thank the reviewer for the careful consideration of our manuscript and the valuable suggestions for improvement.*

1) The authors should provide a model validation in neonates and infant for plasma concentrations which should be similar to the model validation for adults and children in Figure 1. Previously published concentration time profiles could be found in the following publications:

Pokela ML, Olkkola KT, Seppala T, Koivisto M. Age-related morphine kinetics in infants. Dev Pharmacol Ther. 1993;20(1-2):26–34.
Olkkola KT, Maunuksela EL, Korpela R, Rosenberg PH. Kinetics and dynamics of postoperative intravenous morphine in children. Clin Pharmacol Ther. 1988;44(2):128–136

A validated systemic concentration time profile is the prerequisite for the development of brain compartments for CSF concentrations. Without these validations, it is difficult to evaluate the reason for the biased model prediction in Figure 1D.

*We agree that an extra validation step of the plasma concentrations in neonates would benefit the model before making predictions on drug concentrations in CSF, like we did for adults and older children. The problem with the references suggested is that we cannot be sure that they fully reflect the population from which in our study CSF samples were obtained (Fig 2B, revised manuscript). A match/mismatch between simulated and measured plasma drug concentrations for these studies is therefore not very informative. Morphine clearance and Vss are vital parameters to predict neonatal plasma PK. We used a study by Wang et al. [1], which consists of a large analysis of multiple neonatal and pediatric studies and could be considered to contain “typical” PK parameters for the neonatal and pediatric population. We therefore prefer using this “typical” clearance value by Wang et al. and the PBPK model estimated Vss (which is close to the value by Wang et al.).
A clear limitation of the CSF dataset we use is that no paired plasma samples were obtained. This would help to better evaluate the model prediction in Figure 2B, which indeed could be due to a biased plasma PK prediction as well as brain morphine disposition. In our discussion section we now further highlight the uncertainty in neonatal CSF predictions.*

*The following text was added to the discussion section, page 11, lines 257-259 (in the revised document):* “*The model developed in this study has some important limitations and assumptions.* *First, no matching plasma morphine or M6G concentrations were available for the neonatal population, which would be valuable to assess the reason for the slight underprediction of measured CSF values.”*

2) In this manuscript, pain scores were evaluated in healthy volunteers, oncology patients, and neonates. Different studies accessed different types of pain with different clinical instruments. It might not be possible or reasonable to model the different pain scores using the same PD model for different pain instruments and types of pain. Also, the authors did not discuss the pharmacodynamic development in pediatrics. If pediatrics responded to opioids differently throughout postnatal development, it may also support a different pain model for neonates (e.g. Journal of Neuroscience 1 April 1998, 18 (7) 2538-2549 and PAIN155 (2014) 168–178169). The authors should also notice that the model predicted concentrations in Figure 1D were lower than the observed concentrations, which might be another reason for the underestimated response in neonates in Figure 2.

*As pointed out by the reviewer, pain scores measured with different clinical instruments can result in heterogeneity of PD measurements. Ideally, we would make use of control studies in which an experimental pain stimulus was given in all populations with and without morphine, however, this is only possible in healthy adult volunteers but not in pediatric populations due to ethical limitations. The suggestion that in neonates a quantitatively different PD relationship might be more suitable, is consistent with our findings in Figure 3C (panel for neonates and young children), as observed effects were largely underpredicted with the current PD model. In the discussion section we now further substantiate this finding with data from pre-clinical literature, in which increased sensitivity to morphine was described. This confirms the conclusion that developmental differences in PD are responsible for the increased sensitivity to morphine in neonates. Future studies should clarify the age-dependent changes in neonatal PD and incorporate them into the model.*

*The following text was added to discussion section, pages 10-11 lines, 236-245:* *“**In line with increased neonatal sensitivity observed here, increased sensitivity to MOR agonists has been described in rats of the same age group. Spinal injection of [D-Ala2, N-Me-Phe4, Gly5-ol] enkephalin (DAMGO) in 10 days postnatal rats, or epidural injection of morphine in 3 days postnatal rats, resulted in more pronounced analgesia compared to effects seen in older pediatric and adult rats [2, 3]. This could be explained by higher neonatal MOR expression in large diameter dorsal root ganglia and dorsal horn, which are important in relaying pain signals to the central nervous system [4-6]. In addition, descending pain pathways do not appear to be mature at birth [2, 7]. More elaborate PD models can be considered in the future, accounting for age-related differences in MOR expression and morphine binding-effect relationship [8].”*

*We agree with the reviewer that the underprediction of CSF values in Figure 2B could, theoretically, be an explanation for the underestimation in the response seen in Figure 3C, left panel. To evaluate this, we performed a simulation for the theoretical situation with a 50% lowered CL for neonates, which resulted in an optimized prediction of CSF concentrations (see adjusted Figure 2B below). However, it hardly influenced the PD model output in Figure 3C (see Figure below),* *indicating that the underestimation in CSF values was not the cause of the underestimated response in neonates. These figures have been included in supplementary Fig 4.*

**Fig. 2B**

**Fig. 3C**

*Please see for comparison the original Fig. 3C (without 50% CL reduction) below, as included in main text of manuscript.*

*The following text was added to the results section, page 8, lines 181-183: “The underestimation in CSF values (Fig 2B) was not the cause of the overestimated pain scores in neonates (Fig 3C), which was investigated using a simulation with 50% of the original neonatal clearance (supplementary Fig 4).”*

Minor

1) Since the transporter has an impact on distribution, the authors should also demonstrate that the morphine distribution (Vss) is reasonably estimated in adults and children. Although volume of distribution is estimated based on chemical properties in a PBPK model, the authors should provide the estimation method and the estimated volume of distribution for morphine and M6G. Also, these estimated values should be close to the previously published steady state volume of distribution for morphine after IV administration of morphine (e.g. Clin Pharmacol Ther. 2002 Aug;72(2):151-62) and for M6G after IV administration of M6G (e.g. Acute Pain (2006) 8, 63—71).

*As is common in PBPK model development, tissue partitioning coefficients (Kps) are calculated based on the Rodgers and Rowlands algorithms, which we refer to in the publication and used in a previous modelling paper [9]. In addition, Kps are used in the Simcyp and PKsim PBPK software packages regularly. Predicted Kps in our model were checked against the predicted values in the Simcyp simulator. Multiplication of tissue partitioning coefficients with volumes of the major organs in the PBPK model result in the volume of distribution at steady state. For morphine a Vss of 330L was calculated and 30L for M6G, which is close to the value of 304.1L for morphine and 13.2L and 16.6L for M6G, as reported in the publications suggested by the reviewer. Optimizing M6G Vss to 15L by using a Kp-scalar of 0.5 resulted in a slightly better fit of the observed data to the model predictions and is now incorporated in the model.*

*The following text was added to the method section, page 14, lines 331-335: “In addition, organ partition coefficients were derived using methods described by Rodgers et al., taking into account age-appropriate tissue composition, lipophilicity and ionization of compounds [10, 11]. This method resulted in adult morphine and M6G Vss values of 330L and 30L, respectively. M6G Kp values were optimized using a kp-scalar of 0.5 to better reflect previously published values [12].”

Of note: Due to the minor contribution of morphine BBB Pgp activity on brain drug concentrations and because only Pgp activity in the brain and not any other organ was incorporated, the transporter function hardly affects plasma PK and volume of distribution (please see supplementary fig. 1).*

2) Rstudio (Version 1.1.442) is a GUI not a software or package. In the manuscript, the authors should specify the R version and the packages that were used to develop the PBPK model.

*We apologize for the confusion. The R version used is 3.6.2 and the packages used are: ”deSolve” and “MASS”.
On page 14, line 323 we included: “The model was developed in R Version 3.6.2 with packages “deSolve” and “MASS” installed…”*

3) It will be easier to read if the unit of ng/mL and decimal values were used on y-axis for morphine and M6G concentrations in Figure 1.

*Units in Figure 1 have been changed and decimal values are now used.*

4) In Figure 2, three out of the six figures used the observed individual data to evaluate the model predictions. However, it is difficult to tell the central tendency of the individual data. Please use mean +/- standard deviation to replace the individual data points.

*The individual data points have been changed to mean +/- s.d. for one additional panel in Figure 3, revised manuscript. Unfortunately, the data of the two remaining panels are not gathered at the same points in time, which does not allow to calculate means and standard deviations without pooling of data points over arbitrary time intervals. Therefore, we have decided not to change these.*

**Reviewer #2:** The present work addresses an existing knowledge gap related to the understanding of the underlying sources of morphine response variability in pediatric patients. The authors present an application of PBPK modeling utilizing IVIVE with an incorporated PD model to assess the contribution of Pgp activity and simulated MOR receptor occupancy towards age-related variable morphine response. I find that the work has merit, owing to the relatively novel application of PBPK modeling with additional mechanistic insight of morphine PD for addressing a significant issue in pediatric health care. In current drafting, the primary weaknesses of the work stem from a want for technical details related to the application of in vitro data and apparent absence of the supplemental methods section. My recommendations for major and minor corrections are detailed below:

*We thank the reviewer for the supportive comments and for recognizing the value of our work. In addition, the suggestions for improvement of the manuscript are much appreciated.*

Major comments:

1. The supplementary methods section S1 appears to be missing from the document on page 21 – there is a subsection title, but no subtext beneath it prior to the references subsection. The supplied hypertext link included on the final page of the pdf document leads to a download of the supplemental figure only, and no additional source files appear on the reviewer task page. The supplementary methods for morphine and M6G C/E relationship will need to be reviewed prior to rendering a final disposition on this manuscript.

*We apologize for the confusion we may have caused. The equations belonging to the figure in the supplement, including the references used to parameterize the formulas, were described in the main body of the manuscript in the methods section under: “PD model development” (pages 17-18, lines 373-401 in the revised document). We have re-named “Supplementary methods section S1” in the current version of the manuscript to: “Supplementary Fig. 5”. For clarification, we also included the equations underlying the concentration-effect relationship in the supplementary figure.*

2. A primary conclusion of the study is that Pgp is unlikely to play a significant role in age-related, variable morphine response. Notably, assumptions are made in the application of IVIVE of Pgp activity from cell culture-derived data, and detail is also missing from discussion of the in vitro methods. This makes it difficult to determine the validity of the conclusion. It is understandably out of reach for the typical wet lab to quantitate enzyme expression levels using advanced MS-based proteomics, but ideally there should be some methodology in place to ensure standardization, and if feasible, a qualitative assessment of expression levels (e.g. western blot against a known standard). The main concern here is that the literature range of 0.19 – 7.02 pmol/mg protein seems quite wide, and in the Methods section it is only discussed that 0.19 pmol/mg was initially investigated. Specific questions I have regarding this application include:
a. Was the full range of 0.19 – 7.02 pmol/mg abundance of Pgp investigated, and were model sensitivity analyses done on this parameter? If the model results were not significantly different across this range, or if values above 0.19 pmol/mg produce unreasonable model performance then it should be stated as such in the text.

*The reviewer raises a valid point and this issue requires further explanation. The in vitro Pgp expression used in this study is indeed the lowest reported value of 0.19 pmol/mg, as this abundance is inversely proportional to the BBB efflux clearance according to eq. 3 (*$CLefflux,vivo=CLefflux, vitro*\frac{abundance \left( ex vivo \right)}{abundance \left( in vitro \right)}*BMvPGB*BW)$*, resulting in the highest transporter-mediated activity in the model. As mentioned by the reviewer, we conclude that even in this situation Pgp has a (relatively) minor role in the description of morphine PK and PD, compared to a complete absence of Pgp-mediated morphine activity in the BBB. In other words, reasonable model performance could also be obtained without inclusion of Pgp, indicating that the model is not very sensitive to this parameter. Values higher than 0.19 pmol/mg in the reported age range will have a smaller effect on PK and PD profiles and, like a situation without Pgp, will also result in a reasonable but not better model performance. To provide proof for these statements a sensitivity analysis was added to the supplementary material (suppl. Fig. 1).*

*The following text was added to the results section, page 6, lines 138-140: “**Due to the moderate sensitivity of model output to the range in reported in vitro MDCK-Pgp expression values, this was maintained at 0.19 pmol/mg (see methods section and supplementary Fig. 1).”, and*

*Page 9, line 198-203: “**To quantify the sensitivity of the model to: (1) in vitro Pgp transporter expression and (2) the morphine equilibrium dissociation constant, sensitivity analyses were performed (supplementary Fig. 1 and 2). These parameters were evaluated over the wide range of measured values that have been reported in literature (see methods section). The model appeared only moderately sensitive to variation in in vitro Pgp transporter expression values, while being sensitive to variability in the morphine equilibrium dissociation constant.”*

b. If the model predictions are sensitive across this range, it will be useful to know if cell culture conditions were consistent between experiments and between controls of individual experiments. The most salient variable in this regard should be cell passage number after reviving cells from cryo-storage. Please add any known relevant details regarding how cell culture conditions were controlled.

*As indicated above, the model is not very sensitive to the morphine BBB active transport parameter. Nevertheless, cell culture was performed under the same conditions with passage numbers between P15 and P23. In addition, the results of the individual experiments (N=3) with morphine and digoxin are relatively consistent with net efflux ratios of 1.44, 1.39, 1.07 and 1.8, 5.7, 3.6, respectively, which all point to active efflux. Experiments were performed in HBSS plus 10mM HEPES without albumin, to prevent drug protein binding.*

*The following text was added on page 13, lines 295-296: “…were seeded with MDCKII-Pgp cells (**3*10^4 cells at 9*10^4 cells/cm2, passage number 15-23)…”*

c. If any of the above conditions constitutes a limitation on interpreting the Pgp contribution to model predictive performance, please include a brief statement in the Discussion section.

*We believe that our approach, in which we included a parameter value that resulted in highest scaled morphine Pgp activity in the BBB, now complemented with the suggested sensitivity analysis, acknowledges the uncertainty caused by the wide range of reported transporter abundances. Even under the condition of highest P-gp activity in the model, the transporter contribution to morphine ECF exposure is limited. Therefore, we concluded that Pgp does not play a significant role.*

3. Similar to point 2 above, the data used for MOR ki values for morphine and M6G are average values from tabulated data in reference 23 (Osbourne et al., 2000). Ranges are given on Page 10, Lines 237-8: which derives from 10 prior in vitro studies using seven different tissue models and seven different radiolabeled ligands. The large variability of this data is mentioned in the Discussion section (as it should be) but the current work would benefit from additional commentary on the sensitivity of the PD model predictive performance to ki values. At the very least, I would suggest for median ki values also be investigated/discussed since the distributions for both morphine and M6G are significantly biased and the fold difference between median and average values are between ~3 - 4.

*Model predictions are indeed sensitive to changes in the Ki for MOR. We agree with the reviewer that the wide range of reported values is a reason for concern, which we addressed in the discussion section. The issue raised is important, because the wide variability would prevent development of prospective PD models in case no clinical response data are available to verify model performance. To further consider and visualize this issue, we performed a sensitivity analysis where we explored model performance over the range of reported values and added this in supplementary Fig 2.*

*The text added to the manuscript described under the reviewers’ major comment 2a also addresses this reviewers’ comment (page 9, lines 198-203 in the revised manuscript).*

*In addition, the following text was added to the discussion section, page 11, lines 264-266: “This is an important limitation, as model outputs proved sensitive to this parameter, which would hamper prospective model development if no clinical PD data is available to select the optimal value.”;*

*and to the methods section, page 18, lines 382-384: “**Equilibrium dissociation constants for MOR have been reviewed previously and average values of 11.8 nM for morphine and 42.5 nM for M6G were used, as this resulted in the best fit of the model to measured adult PD data*.”

Requests for minor corrections:

1. Page 4, line 82: I suggest using a more specific term in place of “drug efficacy” here. I would think to simply say “pharmacodynamics” though I’m unsure if that captures your intent.

*Drug efficacy is changed into pharmacodynamics
On page 4, line 81-82 we added: “Currently, it is not known whether this results from age-related changes in brain tissue pharmacokinetics or drug pharmacodynamics.”*

2. Page 4, line 90-91: I missed a reference for a developmental increase in brain Pgp activity as this does not appear to be covered by reference 13 (Schinkel et al., 1995)

*The reference by Schinkel et al. indeed only reports the fold-increase in brain exposure. Another reference by Tsai et al. 2002 [13] covering the developmental increase in Pgp expression, has now been included.*

3. Page 5, line 100: reference 18 as listed in the bibliography also does not appear to support the statement of successful application of IVIVE to transporter abundance/scaling as it only discusses abundance of Phase-II UGT family enzymes.

*The reference by Bhatt et al. was removed and reference by Emoto et al. [14] was included. In this study developmental OCT1 liver expression has been incorporated in a morphine PBPK model.*

4. Page 6, Lines 134-136: (Figure 1B) The model appears somewhat biased towards underpredicting peak, child CSF concentration for morphine. While I don’t think this is a huge problem, I was curious about the correlation to the PD model performance (Figure 2C) which may appear to overpredict morphine effect. Do the authors have any thoughts?

*Although we agree that the peak CSF concentrations appear underpredicted, it does not have a large influence on PD model performance. When lowering the clearance in the model by 60% to increase CSF concentrations, the analgesic response was hardly affected as can be seen in the figures below.*

*In addition, although CSF concentrations appear slightly underpredicted, we want to emphasize that this is not the case for the model predictions of ECF concentration in this age range (figure 2A, revised manuscript, see below, no changes to drug clearance included). This could be considered even more valuable, as brain ECF concentrations feed directly into the PD model.*

5. Page 6, Line 135: text mentions “drug concentrations”, but should probably also list “metabolite”

*Thanks for pointing out this error. The metabolite (M6G) is now included on
page 6, line 135-137:* “*Simulations reasonably captured measured morphine and M6G concentrations in plasma and CSF for adults and children.”*

6. Page 7, Line 168: No variability was included for the PD model parameters. (see major comment 3 above)

*The reported dissociation constant values show a large variation due to a.o. the differences in experimental setup and different compounds used. Mean values of 11.8 nM form morphine and 42.5 nM for M6G were used in our model as point estimates. The variability has now been explored in a sensitivity analysis, as described also under the reviewer’s major comment 3 (and is shown below). Indeed, the large parameter variability would hamper development of prospective models if no clinical PD data is available for model calibration.*

*The text added to the manuscript described under the reviewers’ major comment 2a also addresses this reviewers’ comment (page 9, lines 198-203 in the revised manuscript).*

*The text added to the manuscript described under the reviewers’ major comment 3 also addresses this reviewers’ comment (page 11, lines 264-266 in the revised manuscript).*

7. Page 12, Line 271: please provide also the cell-seeding density (ie 3*10^4 cells at 9.1*10^4 cells/cm^2)

*Cell seeding density is now provided on
page 13 line 296: “…(3*10^4 cells at 9*10^4 cells/cm2, passage number 15-23)…”*

8. Page 13, Line 297: Please list the R packages and any specific libraries used for PBPK model development, construction and validation.

*R packages “deSolve” and “MASS” were used for this work and this information has been included in the methods section on*
*page 14 line 323: “The model was developed in R Version 3.6.2 with packages “deSolve” and “MASS” installed…”*

9. Page 19, line 405: Text here lists range for pediatric/adolescent pain scores as 0 to 3, yet Figure 2C has data points at 3.5 and 4.

*This inconsistency has now been resolved on page 20, line 435: “Pain was scored on a scale from 0 (no pain) to 4 (severe pain).”*

**Reviewer #3:** Given unexpected time constraints, I unfortunately did not have the time to review this manuscript with the thoroughness it would merit, and I kindly ask the editor to consider the lack of time to conduct an in-depth review in his final assessment.
The manuscript addresses an important topic, though, and appears to be adequately written.

*We would like to thank the reviewer for the consideration of our work. We feel that, together with the comments of reviewer 1 and 2, it has resulted in a stronger manuscript.*

Minor comments:

Introduction

“The effect of a drug relates more closely to its tissue rather than plasma concentration, particularly for more hydrophilic, permeability-limited compounds, or drugs subject to transporter-mediated transfer” – I believe this statement is not true; the effect of a drug rather relates to the drug concentrations at the target site. The drug target can also be in the blood (e.g. anticoagulants) or in the GI lumen (e.g. antacids, some anthelmintics). I suggest rephrasing.

*We agree with the reviewer that this statement is incomplete. We rephrased the following text on page 4, lines 83-85 in the revised manuscript:* *“The effect of a drug relates to its concentration at the target site. Disposition into organs can be hampered particularly for more hydrophilic, permeability-limited compounds, or drugs subject to transporter-mediated transfer.”*

“PBPK models combine knowledge on physiological processes and drug-specific properties in a multi-compartmental structure, which make them less dependent on clinically measured values compared to popPK models” This is also a very bold statement; please be more precise: PBPK predictions are less dependent on clinical data, but in many cases you need these data nonetheless to evaluate the model. Otherwise the value of the predictions cannot be assessed. I suggest rephrasing.

*We added the following sentence to highlight the importance of clinical data for model verification on page 5, lines 105-109:*

*“PBPK models combine knowledge on physiological processes and drug-specific properties in a multi-compartmental structure, which make them less dependent on clinically measured values compared to popPK models. … Currently, however, clinical data are still is needed for model verification or optimization.”*

“Another source of variability in morphine efficacy could originate from…” What is the first source? Maybe rephrase as “Variability in morphine efficacy could originate from…”

*The first source has now been highlighted specifically in the following sentence on page 5, lines 110-111: “Apart from BBB Pgp transporter expression differences, another source of variability in morphine efficacy could originate from differences in pharmacological response between adults and children.”*

“Currently, mechanistic PK/PD models are scarce both for adults and children” I disagree with this statement. There are certainly more mechanistic PK model published, but I don’t have the impression that mechanistic PD models are scarce.

*We agree with the reviewer and have removed this statement form the text.*

“We hypothesize that age-related differences in morphine effect can be explained by the ontogeny
in Pgp expression and/or differences in pharmacodynamic response upon mu opioid receptor (MOR)
binding” Maybe you could elaborate a bit on the mechanism of altered pharmacodynamic response upon receptor binding. From the next sentence I get the impression that altered PD is exclusively modeled by different tissue concentrations of morphine and morphine-6-glucuronide to the MOR which ultimately would be related again to PK but not to different PD.

*We agree that the hypothesized differences require a more elaborate discussion. We have included a paragraph in the discussion section about the evidence obtained from pre-clinical studies pointing to developmental differences in morphine PD. These studies show increased sensitivity in neonates towards morphine, which is not explained by differences in PK. In our study, we included a simple PD relationship independent of age. However, we observed that in neonates this underpredicts the measured PD response, but not in adults and older children, which is consistent with pre-clinical studies. Future studies should quantify the age-dependent PD model in neonates.*

*We would like to refer to the text added to the manuscript described under major comment 2 from reviewer 1, which also addresses this reviewers’ comment (pages 10-11 lines, 236-245 in the revised manuscript)*

Figure 1: Why are the PE plots below the PK profile plots not given for all PK plots but only for panel A and B?

*In panel A and B (Fig. 1 in the revised version of the manuscript) the measured data were derived from multiple individuals. Inter-individual differences are therefore expected to cancel out in the analysis (see methods). This means that data from an “average” individual were compared to the median simulated PK profiles resulting in the PE plots. The other panels (Fig. 2 in the revised manuscript) present multiple datapoints from only 1-3 individuals, which therefore could systematically lay above/below the median simulated value, and makes these plots less informative.*

Figure 1: Please provide a zoom-in for some panels; the axis limits of some PK profiles are too squeezed, and the plots are therefore not very informative. It may even be better to separate Fig. 1 in multiple figures that have a larger size/resolution.

*Figure 1 has been separated in multiple figures (see Fig. 1 and Fig. 2 in the revised manuscript)*

Figure 1D: Can you also provide the PK profile for the neonates? This will be more informative than the GOF plot.

*A single CSF drug concentration measurement was obtained from different neonates in clinical practice. All neonates received an individualized dosing regimen and had unique timing of CSF collection. Multiple (individualized) simulations were performed, which now have been included in supplementary Fig 3, in addition to the (more concise) GOF plot presented in the main text.

Page 7 line 157: “Individual simulations are shown in supplementary Fig 3.”*

Figure 1E: Please add the unit of the y-axis (mg/L ,I assume)

*Unit of y-axis has been added.*

Can the mismatch between observed and predicted neonatal pain score (Fig. 2C) be fully explained by the relatively poor predictive performance of the neonatal CSF morphine predictions? This question could be further explored through e.g. conducting a sensitivity analysis.

*We would like to refer to major comment 2 from reviewer 1, which also addresses this reviewers’ comment.*

I think the discussion could be improved if the technical aspects of model are more broadly imbedded in what has been published previously by other groups. E.g. how is this model structure and parameterization and the implemented ontogeny different from other models?

*We included on page 9, lines 211-216:* *“The model developed in this study uses the structure proposed by Gaohua et al., which was previously extended by us to incorporate a brain barrier function based on age-related physiological parameters [9, 15]. Other models including brain compartments were built using on rat data and were extrapolated to human [16, 17]. Previous human PBPK models predicted brain concentrations mainly for drugs passing the BBB via passive diffusion, or active transport was translated from animal species by allometric scaling [9, 15, 17, 18].”*

Since the model is developed in R will it be made available to the community upon publication? I think this would be of great value. These days I see too many models that are not available open access to the community and independent reproduction of these models often fails which I believe undermines in the long term the confidence in these approaches.

*We publish the pediatric PBPK/PD model together with this manuscript. The model code is now available in supplementary file 1.*

*We added the following sentence on page 14 lines 326-328:* *“The pediatric morphine model code is provided in supplementary file S1, and can be adjusted to an adult version by using the physiological parameters published previously [26].”*

**References**

1.	Wang C, Sadhavisvam S, Krekels EH, Dahan A, Tibboel D, Danhof M, et al. Developmental changes in morphine clearance across the entire paediatric age range are best described by a bodyweight-dependent exponent model. Clin Drug Investig. 2013;33(7):523-34. Epub 2013/06/12. doi: 10.1007/s40261-013-0097-6. PubMed PMID: 23754691.

2.	Kwok CH, Devonshire IM, Bennett AJ, Hathway GJ. Postnatal maturation of endogenous opioid systems within the periaqueductal grey and spinal dorsal horn of the rat. Pain. 2014;155(1):168-78. Epub 2013/10/01. doi: 10.1016/j.pain.2013.09.022. PubMed PMID: 24076162; PubMed Central PMCID: PMCPMC3894430.

3.	Marsh D, Dickenson A, Hatch D, Fitzgerald M. Epidural opioid analgesia in infant rats I: mechanical and heat responses. Pain. 1999;82(1):23-32. Epub 1999/07/28. doi: 10.1016/s0304-3959(99)00028-7. PubMed PMID: 10422656.

4.	Beland B, Fitzgerald M. Mu- and delta-opioid receptors are downregulated in the largest diameter primary sensory neurons during postnatal development in rats. Pain. 2001;90(1-2):143-50. Epub 2001/02/13. doi: 10.1016/s0304-3959(00)00397-3. PubMed PMID: 11166980.

5.	Nandi R, Fitzgerald M. Opioid analgesia in the newborn. Eur J Pain. 2005;9(2):105-8. Epub 2005/03/02. doi: 10.1016/j.ejpain.2004.05.005. PubMed PMID: 15737795.

6.	Rahman W, Dashwood MR, Fitzgerald M, Aynsley-Green A, Dickenson AH. Postnatal development of multiple opioid receptors in the spinal cord and development of spinal morphine analgesia. Brain Res Dev Brain Res. 1998;108(1-2):239-54. Epub 1998/08/07. doi: 10.1016/s0165-3806(98)00054-6. PubMed PMID: 9693800.

7.	Hathway GJ, Vega-Avelaira D, Fitzgerald M. A critical period in the supraspinal control of pain: opioid-dependent changes in brainstem rostroventral medulla function in preadolescence. Pain. 2012;153(4):775-83. Epub 2012/02/14. doi: 10.1016/j.pain.2011.11.011. PubMed PMID: 22325744; PubMed Central PMCID: PMCPMC3657184.

8.	Ueno E, Liu DD, Ho IK, Hoskins B. Opiate receptor characteristics in brains from young, mature and aged mice. Neurobiol Aging. 1988;9(3):279-83. Epub 1988/05/01. doi: 10.1016/s0197-4580(88)80066-6. PubMed PMID: 2840590.

9.	Verscheijden LFM, Koenderink JB, de Wildt SN, Russel FGM. Development of a physiologically-based pharmacokinetic pediatric brain model for prediction of cerebrospinal fluid drug concentrations and the influence of meningitis. PLoS Comput Biol. 2019;15(6):e1007117. Epub 2019/06/14. doi: 10.1371/journal.pcbi.1007117. PubMed PMID: 31194730; PubMed Central PMCID: PMCPMC6592555.

10.	Rodgers T, Rowland M. Physiologically based pharmacokinetic modelling 2: predicting the tissue distribution of acids, very weak bases, neutrals and zwitterions. J Pharm Sci. 2006;95(6):1238-57. Epub 2006/04/28. doi: 10.1002/jps.20502. PubMed PMID: 16639716.

11.	Rodgers T, Leahy D, Rowland M. Physiologically based pharmacokinetic modeling 1: predicting the tissue distribution of moderate-to-strong bases. J Pharm Sci. 2005;94(6):1259-76. Epub 2005/04/29. doi: 10.1002/jps.20322. PubMed PMID: 15858854.

12.	Lötsch J, Skarke C, Schmidt H, Liefhold J, Geisslinger G. Pharmacokinetic modeling to predict morphine and morphine-6-glucuronide plasma concentrations in healthy young volunteers. Clin Pharmacol Ther. 2002;72(2):151-62. Epub 2002/08/22. doi: 10.1067/mcp.2002.126172. PubMed PMID: 12189362.

13.	Tsai CE, Daood MJ, Lane RH, Hansen TW, Gruetzmacher EM, Watchko JF. P-glycoprotein expression in mouse brain increases with maturation. Biol Neonate. 2002;81(1):58-64. Epub 2002/01/23. doi: 10.1159/000047185. PubMed PMID: 11803178.

14.	Emoto C, Johnson TN, Neuhoff S, Hahn D, Vinks AA, Fukuda T. PBPK Model of Morphine Incorporating Developmental Changes in Hepatic OCT1 and UGT2B7 Proteins to Explain the Variability in Clearances in Neonates and Small Infants. CPT Pharmacometrics Syst Pharmacol. 2018;7(7):464-73. Epub 2018/06/20. doi: 10.1002/psp4.12306. PubMed PMID: 29920988; PubMed Central PMCID: PMCPMC6063742.

15.	Gaohua L, Neuhoff S, Johnson TN, Rostami-Hodjegan A, Jamei M. Development of a permeability-limited model of the human brain and cerebrospinal fluid (CSF) to integrate known physiological and biological knowledge: Estimating time varying CSF drug concentrations and their variability using in vitro data. Drug Metab Pharmacokinet. 2016;31(3):224-33. Epub 2016/05/30. doi: 10.1016/j.dmpk.2016.03.005. PubMed PMID: 27236639.

16.	Yamamoto Y, Välitalo PA, Huntjens DR, Proost JH, Vermeulen A, Krauwinkel W, et al. Predicting Drug Concentration-Time Profiles in Multiple CNS Compartments Using a Comprehensive Physiologically-Based Pharmacokinetic Model. CPT Pharmacometrics Syst Pharmacol. 2017;6(11):765-77. Epub 2017/09/12. doi: 10.1002/psp4.12250. PubMed PMID: 28891201; PubMed Central PMCID: PMCPMC5702903.

17.	Yamamoto Y, Valitalo PA, Wong YC, Huntjens DR, Proost JH, Vermeulen A, et al. Prediction of human CNS pharmacokinetics using a physiologically-based pharmacokinetic modeling approach. Eur J Pharm Sci. 2018;112:168-79. Epub 2017/11/15. doi: 10.1016/j.ejps.2017.11.011. PubMed PMID: 29133240.

18.	Li J, Wu J, Bao X, Honea N, Xie Y, Kim S, et al. Quantitative and Mechanistic Understanding of AZD1775 Penetration across Human Blood-Brain Barrier in Glioblastoma Patients Using an IVIVE-PBPK Modeling Approach. Clin Cancer Res. 2017;23(24):7454-66. Epub 2017/09/21. doi: 10.1158/1078-0432.Ccr-17-0983. PubMed PMID: 28928160; PubMed Central PMCID: PMCPMC6097184.
