# Supplementary material for: Physiologically based pharmacokinetic/pharmacodynamic model for the prediction of morphine brain disposition and analgesia in adults and children
Source: PLoS Comput Biol. 2021 Mar 4;17(3):e1008786. doi: 10.1371/journal.pcbi.1008786 (PMC7963108; doi:10.1371/journal.pcbi.1008786)
Supplement: Attachment — Submitted filename: Response to reviewer_Verscheijden.docx [file pcbi.1008786.s008.docx]

Reviewer #1: The authors have addressed almost all the comments I had previously except for my major comment #1. The authors' simulation for neonatal CSF concentration addressed part of the concern on pharmacodynamic response. However, I still have the following comment regards to the same request I had previously:

The external evaluation for plasma concentration in neonates is not for the explanation of biased pharmacodynamic prediction but also for the evaluation of PBPK model. The authors collected clearance model from different publications (e.g., Wang et al.) for morphine and M6G and forced it into the PBPK model. The original model assumptions and misspecifications may not remain validate in the new PBPK model. In the response to my major comment 1, the authors simply claimed that they cannot be sure that the recommend external data fully reflect the population from which the authors collected in this study. However, the author did not explain what the difference between these two populations is and how it may lead to a different PK characteristic. The authors claim seems to be bold. Unless there's specific disease related factors that may change PK, I would believe PK should be close enough in different patient populations. Therefore, I still believe that the authors should provide the external evaluation of neonatal plasma concentration profile. I recommend the authors follow the external evaluation step in the following article to evaluate their PBPK model.

Liu et al. Mechanistic population pharmacokinetics of morphine in neonates with abstinence syndrome after oral administration of diluted tincture of opium. The Journal of Clinical Pharmacology 56(8).

Particularly, in this publication, it showed how one integrated morphine PK model could describe PK across different age groups as an external evaluation. The two popPK model cited (Wang and Bouwmeester) in the current manuscript are both included in this publication. Morphine population pharmacokinetics model and PBPK model have been published previously many times. I do believe we should have a better model in the following publications, and an external evaluation can tell us directly how these models work in different aspects.

*Thanks again to the reviewer for the careful evaluation of our manuscript and pointing out the necessity to also validate the PBPK model with external data. We apologize that we did not fully address this comment in the revised version.*

*We have now compared our model simulations in plasma with the neonatal data reported by Pokela et al. (1993) that is referred to in the publication by Liu et al. (2016), as suggested by the reviewer (1, 2). Predicted and observed plasma PK data have been included in Figure 2B. We found that simulations in neonatal plasma slightly underpredicted observed data.*

*We do not believe that this underprediction justifies changing the parameters included our model, because the study by Pokela et al. is small, which potentially results in an imprecise estimate of plasma PK. The paper by Liu et al. also included the model by Knibbe et al. (3), which underpredicted Pokela’s plasma concentrations consistent with our results, while this model did not show biased predictions in another external validation study (4). This indicates that the measured values by Pokela et al. might be at the high side and differences between study populations exist. Another explanation for this difference could be the relatively old HPLC analysis technique used by Pokela et al. No other single dose studies are currently available for validation of plasma drug predictions. However, we agree that further external validation and optimization by Wang et al. and others might result in improved models, more predictive for all datasets.*

*The focus of the current study was, however, the prediction of brain and CSF drug concentrations and resultant pharmacodynamics. In our opinion the neonatal PBPK-PD model can be used to confirm the increased sensitivity of neonates to morphine, especially when optimization is performed as shown in supplementary figure 4.*

*The following text was added to the methods section, page 20, lines 430-432:*

*“Due to the absence of paired plasma data in our cohort, the neonatal plasma model output was compared with mean measured plasma concentrations from Pokela et al., similar to what has been described previously [30, 91].”*

*The following adjustments were made in the results section, page 7, lines 154-159:*

*“In the neonatal population, mean observed plasma concentrations were slightly underpredicted when clearance was not optimized (Fig 2B). For a separate group of neonates and young children who underwent CSF sampling (no paired plasma samples available) in which dosing regimens were variable, individual simulations were performed and compared with measured CSF data. Our model slightly underpredicted measured CSF concentrations with a mean prediction error (PE) of 0.41 (Fig 2C). Individual simulations in CSF are shown in supplementary Fig 3.*

**Reference**

1.	Pokela ML, Olkkola KT, Seppälä T, Koivisto M. Age-related morphine kinetics in infants. Dev Pharmacol Ther. 1993;20(1-2):26-34.

2.	Liu T, Lewis T, Gauda E, Gobburu J, Ivaturi V. Mechanistic Population Pharmacokinetics of Morphine in Neonates With Abstinence Syndrome After Oral Administration of Diluted Tincture of Opium. J Clin Pharmacol. 2016;56(8):1009-18.

3.	Knibbe CA, Krekels EH, van den Anker JN, DeJongh J, Santen GW, van Dijk M, et al. Morphine glucuronidation in preterm neonates, infants and children younger than 3 years. Clin Pharmacokinet. 2009;48(6):371-85.

4.	Krekels EH, Tibboel D, de Wildt SN, Ceelie I, Dahan A, van Dijk M, et al. Evidence-based morphine dosing for postoperative neonates and infants. Clin Pharmacokinet. 2014;53(6):553-63.
